# 肺癌硬膜下脊髓外转移患者临床特征分析

**DOI:** 10.3779/j.issn.1009-3419.2016.08.10

**Published:** 2016-08-20

**Authors:** 燕 徐, 巍 钟, 静 赵, 闽江 陈, 龙芸 李, 孟昭 王

**Affiliations:** 100730 北京, 中国医学科学院, 北京协和医学院, 北京协和医院呼吸内科 Department of Respiratory Disease, Peking Union Medical College Hospital, Chinese Academy of Medical Science and Peking Union Medical College, Beijing 100730, China

**Keywords:** 肺肿瘤, 脑膜转移癌, 脊髓, 硬膜下脊髓外转移, Lung neoplasms, Leptomeningeal metastasis, Spinal cord, Intradural extramedullary metastases

## Abstract

**背景与目的:**

肺癌硬膜下脊髓外转移罕见, 可导致严重的神经损害, 本研究旨在阐明其临床特征。

**方法:**

2013年5月-2016年5月, 北京协和医院8例确诊硬膜下脊髓外转移肺癌患者纳入该研究, 系统回顾分析临床资料。

**结果:**

7例非小细胞肺癌及1例小细胞肺癌合并硬膜下脊髓外转移。马尾综合征是最常见的临床表现。行腰椎穿刺的5例(100%)患者脑脊液找到肿瘤细胞。脊髓增强核磁(magnetic resonance imaging, MRI)发现, 3例软脊膜弥漫线样增强, 4例硬膜下脊髓外多发结节, 1例具有上述两种表现。4例接受靶向治疗和/或放疗患者神经系统症状改善或稳定。中位生存时间是5.8个月。

**结论:**

硬膜下脊髓外转移需依靠神经系统症状及增强MRI影像学检查诊断。靶向治疗和/或放疗可能有效。

## Abstract (en)

**Background and objective:**

Intradural extramedullary spinal cord metastases in lung cancer is rare, and it leads to severe neurological damage.The aim of this study is to identify the clinical features of intradural extramedullary spinal cord metastases in primary lung cancer patients.

**Methods:**

The 8 cases of lung cancer with intradural extramedullary metastases, who were hospitalized in Peking Union Medical College Hospital (PUMCH) during May 2013 to May 2016, were enrolled in the retrospective study.Medical charts of the 8 patients were reviewed systematically.

**Results:**

Intradural extramedullary spinal cord metastases was diagnosed in 7 cases with non-small cell lung cancer (NSCLC) and 1 case with small cell lung cancer (SCLC).Cauda equina syndrome was the most common clinical manifestation.Malignant cells in cerebrospinal fluid were positive in all the 5 cases (100%) who underwent lumbar puncture.Contrast-enhanced magnetic resonance imaging (MRI) of spine manifested as diffuse abnormal enhancement of pial lining of spinal cordin 3 cases, intradural extramedullary nodules in 4 cases, and both of them in 1 case.Neurological symptoms were improved or stable in 4 cases who underwent targeted therapy and/or radiotherapy.The median overall survival was 5.8 months.

**Conclusion:**

Intradural extramedullary spinal cord metastases can be diagnosed with caution according to its neurological symptoms and contrast-enhanced MRI presentation.Targeted therapy and/or radiotherapy may be effective for symptoms control.

晚期肺癌(lung cancer, LC)合并转移癌脊髓压迫症(metastatic spinal cord compression, MSCC)是一种特殊的临床表现, 临床症状重, 诊断困难, 预后极差。依据肺癌转移病灶与脊髓周围解剖结构的相关性, 可将脊髓受累表现分为三种类型^[1]^:①硬膜外压迫, 包括脊柱骨转移和/或硬膜外肿瘤转移病灶, 转移灶肿瘤直接生长或椎体发生病理性骨折压迫硬脊膜及脊髓, 进而导致的脊髓压迫综合征^[2]^, 导致脊髓受累, 是最常见的类型; ②髓内转移^[3, 4]^:脊髓髓内转移病灶直接压迫脊髓, 导致临床症状; ③硬膜下脊髓外转移(intradural extramedullary spinal cord metastases), 也称为软脊膜转移(spinal cord pial metastases), 是脑(脊)膜转移(leptomeningeal metastasis, LM)相关脊髓受累的一种特殊临床表现^[5, 6]^。

脑(脊)膜转移是一种相对少见的中枢转移, 恶性肿瘤脑(脊)膜转移是恶性肿瘤细胞在脑和脊髓的蛛网膜下腔内弥漫转移, 脑和脊髓的软脑(脊)膜弥漫性或多灶性、局限性肿瘤细胞浸润, 进而引起一系列的临床症状^[7]^。脑(脊)膜转移临床症状多样, 以头痛、恶心、呕吐等颅高压为典型表现的软脑膜转移的患者多见, 然而, 部分患者临床表现脊髓和脊神经根受累的表现^[5, 6]^, 如膀胱和直肠括约肌功能障碍、神经根性疼痛、肌力下降等, 是由于恶性肿瘤在蛛网膜下腔广泛播散, 在脊髓的软脊膜以及脊神经表面形成浸润, 使得脊髓和脊神经受累, 进而引发相应的临床症状, 肺癌软脊膜转移起病相对隐匿, 治疗效果差, 预后极差。

在临床工作中, 肿瘤科医师对于硬膜下脊髓外的相关表现认识度低, 易忽略相关软脊膜转移临床症状, 且即使怀疑存在脑膜转移的临床症状, 进行头颅增强核磁(magnetic resonance imaging, MRI)进行进一步判断, 忽略全脊髓增强MRI, 无法评估软脊膜病变情况。本研究对我院肺癌合并硬膜下脊髓外转移(软脊膜转移)证据的患者, 总结其临床特征, 以期指导临床工作。

## 1 资料与方法

### 1.1 患者资料

以“脊膜转移”或“脊髓受累”及“肺癌”为检索关键词, 于我院病案室及我科脑膜转移数据库进行搜索, 自2013年5月-2016年5月, 共8例患者诊断为肺癌合并硬膜下脊髓外转移。肺癌硬膜下脊髓外转移(软脊膜转移)诊断标准^[6]^:①组织学或细胞学确诊的肺癌; ②脊髓或脊神经受累的临床症状; ③典型的全脊髓增强MRI髓外硬膜下转移的影像学改变; ④脑脊液找到瘤细胞或转移病灶组织病理证实。符合①+②+③为临床诊断, 符合①+②+③+④为病理确诊。本研究中8例患者均符合前3条临床诊断标准, 临床诊断硬膜下脊髓外转移, 其中5例患者完善腰椎穿刺行脑脊液细胞学检测, 脑脊液中找到瘤细胞为病理确诊的硬膜下脊髓外转移。

### 1.2 研究方法

回顾分析收集患者临床信息, 包括:肺癌的诊断时间、病理结果、分期、表皮生长因子受体(epidermal growth factor receptor, EGFR)检测结果(采用ARMS法)、间变性淋巴瘤激酶(anaplastic lymphoma kinase, *ALK*)融合基因检测结果(免疫组化或Fish法), 肺癌治疗, 软脊膜转移发病症状, 发病时间, 脑脊液常规、生化、细胞学检测结果, 头增强及全脊柱增强MRI影像学表现, 脑(脊)膜转移相关治疗, 及转归。

### 1.3 统计学方法

症状发生时间以患者首次出现脊髓或脊神经受累相关症状开始计算。硬膜下脊髓外转移确诊时间以症状后出现典型脊髓MRI影像学表现时间。OS以硬膜下脊髓外转移诊断的第一天开始计算截止至死亡。患者失访或存活作为删失, 以末次随访时间为时间终点。通过*Kaplan-Meier*法进行计算获得中位生存期(median overall survival, mOS)。计量资料应用Mean±SD表示。计数资料采用率表示。应用SPSS 17.0统计软件进行统计学分析及进行统计学绘图。

## 2 结果

### 2.1 基本临床特征

自2013年5月-2016年5月, 8例患者确诊肺癌合并硬膜下脊髓外(软脊膜)转移(表 1), 其中男性7例, 女性1例, 年龄(53.8±13.4)岁。1例小细胞肺癌, 7例为非小细胞肺癌(non-small cell lung cancer, NSCLC), 其中4例患者检测到*EGFR*突变, 1例患者检测到*ALK*重排, 另2例患者*EGFR*突变及*ALK*重排均为阴性。8例患者中3例患者初次诊断肺癌时行手术治疗, 术后复发。8例患者均曾接受全身系统治疗, 其中5例患者曾接受系统化疗, 有明确驱动基因的5例患者, 4例患者既往曾接受靶向治疗, 其中3例患者为EGFR酪氨酸激酶抑制剂(tyrosine kinase inhibitors, TKIs)治疗过程中确诊软脊膜转移, 1例患者从未应用EGFR-TKIs治疗, 1例*ALK*重排阳性的患者应用克唑替尼治疗后病情进展, 行系统性化疗过程中出现软脊膜转移。8例患者自确诊肺癌至确诊脊膜转移中位时间为:12.8个月(范围:9.1个月-85.2个月)。

**1:** 肺癌合并硬膜下脊髓外转移患者临床特征 Clinical characteristics of lung cancer patients with intradural extramedullary spinal metastasis

ID	Gender	Age	Histology	Driver gene	Time of LM develop (m)	CSF cytology	MRI of the brain and spinal cord			
							BM	LM	Intradural extramedullary nodules	Enhancement of spinal pial lining
1	Male	32	Ad	EGFR Exon 18 G719X	10.0	Y		Y		Y
2	Male	61	Ad	WT	10.5	Y				Y
3	Female	64	Ad	EGFR Exon 21 L858R	43.6	Y				Y
4	Male	41	Ad	EGFR Exon 19 del	85.2	Y	Y		Y	
5	Male	44	Sq	EGFR Exon 21 L858R	20.8	Y	Y	Y	Y	
6	Male	64	Ad	ALK	12.6	NA	Y	Y	Y	Y
7	Male	70	SCLC	NA	12.3	NA	Y		Y	
8	Male	55	Ad	WT	9.1	NA	Y		Y	
MRI:magnetic resonance imaging; Sq:squamous cell carcinoma; Ad:adenocarcinoma; SCLC:small cell carcinoma; WT:wide type; CSF:cerebrospinal fluid; BM:brain metastasis; LM:cerebral leptomeningeal metastasis; m:months; Y:yes; NA:not applicable.										

### 2.2 临床症状

硬膜下脊髓外转移临床症状与受累脊膜病变位置相关, 8例患者有明确腰髓、脊髓圆锥及马尾神经周围脊膜的受累, 因此马尾综合征为其突出表现(8/8, 100%):7例患者尿潴留, 肌力下降7例, 便秘4例, 后背痛或下肢疼痛6例, 行走不稳或无法行走8例, 下肢感觉减退3例; 1例患者有颈髓节段软脊膜病变, 表现为颈肩部疼痛、上肢痛, 上肢肌力下降; 软脑膜受累及颅高压表现也是重要的临床症状, 表现为头痛4例, 头晕6例, 恶心、呕吐4例, 反应迟钝3例, 查体提示脑膜刺激征4例。硬膜下脊髓外转移症状发生至确诊时间51 d(范围:20 d-124 d), 7例患者以硬膜下脊髓外转移相关症状为主要临床表现, 1例患者先诊断脑膜转移, 8个月后出现硬膜下脊髓外转移症状及MRI脊膜受累表现。

### 2.3 脑脊液检测

5例NSCLC患者完成腰椎穿刺行脑脊液检测, 其中3例患者葡萄糖减低, 5例患者蛋白增高, 氯化物无变化, 5例患者(5/5, 100%)脑脊液检测找到肿瘤细胞, 其中*EGFR*突变阳性的4例患者脑脊液中均检测到*EGFR*突变。

### 2.4 全脑及全脊髓MRI检查

8例患者完善头增强MRI及全脊髓增强MRI检查。8例均有明确的腰髓段软脊膜受累及马尾圆锥软脊膜受累。其中1例患者有颈胸髓节段软脊膜受累, 1例患者有胸髓受累表现。3例患者头增强MRI有软脑膜受累表现, 表现为软脑膜线样增强。5例患者合并脑转移表现。软脊膜受累表现为增强核磁见脊髓椎管内脊膜不规则增厚伴线样强化, 或腰椎管内脊髓外T1WI常见结节样软组织信号影, T2WI表现为蛛网膜下腔内大小不一的结节状充盈缺损, 增强扫描多发结节轻度强化。本研究中其中3例患者为软脊膜增厚伴线样强化(图 1), 4例患者表现为椎管内脊髓外结节样改变(图 2), 1例患者同时具有上述两种改变。

**1:**
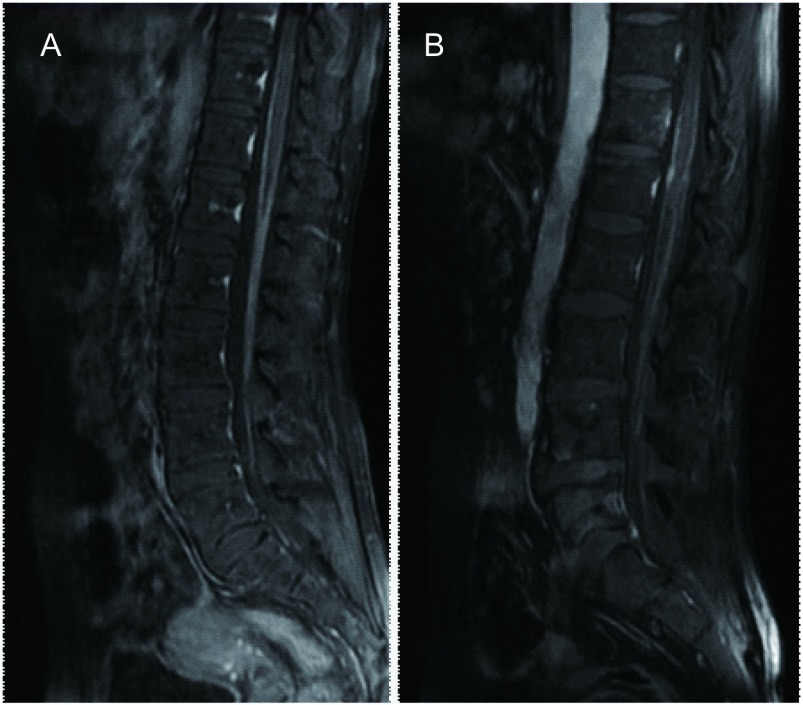
No.3(A)及No.2(B)患者T1压脂腰椎增强MRI表现。腰椎增强MRI显示软脊膜不均匀增厚伴有线样强化。 Sagittal sections of a contrast-enhanced fat-saturated T1-weighted magnetic resonance imaging (MRI) study of the lumbar spinal cord of patient No.2 (B) and patient No.3 (A).Contrast-enhanced MRI of lumbar spine showed diffuse abnormal enhancement of pial lining of spinal cord.

**2:**
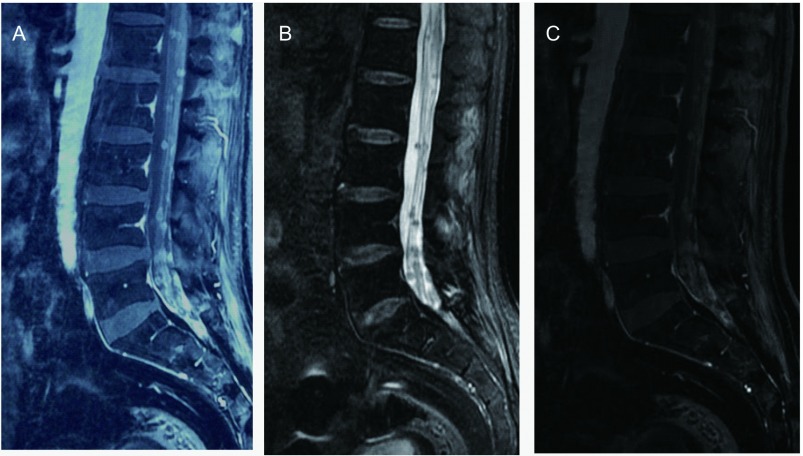
No.7患者腰椎MRI表现。A:T1WI椎管内脊髓外多发稍高信号软组织密度结节; B:T2WI椎管内脊髓外多发低信号的结节样充盈缺损; C:增强MRI显示椎管内脊髓外多发结节轻度强化。 Sagittal sections of MRI study of the lumbar spinal cord of patient No.7.A:T1-weighted imaging showed multiple hyperintense intradural extramedullary nodules; B:T2-weighted imaging showed multiple hypointense intradural extramedullary nodules; C:Contrast-enhanced MRI of lumbar spine showed multiple intradural extramedullary nodules with abnormal enhancement.

### 2.5 治疗与转归

5例NSCLC患者行鞘内注射治疗。4例*EGFR*突变患者行EGFR-TKIs治疗, 但由于该3例患者均为EGFR-TKIs治疗过程中出现硬膜下脊髓外转移, 继续EGFR-TKIs治疗方案(吉非替尼改用厄洛替尼或厄洛替尼改为AZD9291), 患者症状仍无明确改善, No.5号患者无EGFR-TKI治疗史出现软脊膜转移后应用局部放疗及吉非替尼治疗, 症状缓解, 1例患者*ALK*重排, 克唑替尼治疗进展后改为AP方案(培美曲塞+顺铂)化疗, AP方案治疗进展后多西他赛单药治疗过程中发现软脊膜转移, 再次加用色瑞替尼后, 患者症状部分缓解, MRI影像学提示软脊膜病变好转, 2例无突变的NSCLC患者, 曾尝试厄洛替尼治疗, 无效。脊髓病变局部放疗的3例患者, 2例患者症状部分改善, 1例症状稳定。中位生存时间5.8个月(表 2)。

**2:** 硬膜下脊髓外转移患者的治疗结果 Treatment outcomes of patients with intradural extramedullary spinal metastasis

ID	Driver gene	Treatments for peripheral tumor				WBRT	Treatments for intradural extramedullary spinal metastasis					Follow-up	
		Surgery	RT	CT	TKIs		IT	TKIs	RT	Response		Status	OS
1	EGFR Exon 18 G719X	N	N	Y	Y	Y	Y	Y	N	PD		Dead	2.6
2	WT	N	Y	Y	N	N	Y	Y	N	PD		Dead	0.8
3	EGFR Exon 21 L858R	Y	N	N	Y	Y	Y	Y	Y	Stable		Alive	7.0
4	EGFR Exon19del	Y	Y	Y	Y	Y	N	Y	N	PD		Dead	3.9
5	EGFR Exon21 L858R	Y	Y	Y	N	Y	Y	Y	Y	Imporve		Dead	14.0
6	ALK	N	N	Y	Y	N	N	Y	N	Imporve		Alive	4.4
7	--	N	N	Y	N	Y	N	N	N	PD		Missed	0.3
8	WT	N	N	Y	N	N	N	Y	Y	Imporve		Dead	5.8
RT:radiotherapy; CT:chemotherapy; TKIs:tyrosine kinase inhibitors; WBRT:whole brain radiation therapy; OS:overall survival; WT:wide type; PD:progressive disease.													

## 3 讨论

肺癌硬膜下脊髓外(软脊膜)转移是脑(脊)膜转移的一种特殊的临床表现, 肿瘤细胞在脊髓外蛛网膜下腔播散转移, 导致一系列脊髓及脊神经根受累的症状, 表现为转移性脊髓压迫综合症。该罕见转移发生率低, 但患者临床症状重, 一旦发生, 严重影响患者生活质量, 预后极差。本研究总结了8例硬膜下脊髓外转移患者的临床特征和诊治结果, 以期能够指导临床工作。

肿瘤细胞通过血源性播散、局部播散等途径进入蛛网膜下腔, 在脑和脊髓的蛛网膜下腔内弥漫转移, 脑和脊髓的软脑(脊)膜弥漫性或多灶性、局限性肿瘤细胞浸润, 在脊髓表面的软脊膜或脊神经根浸润, 进而引发硬膜下脊髓外(软脊膜)转移相应病变及相应的临床症状。Kizawa等^[5]^分析了实体瘤脑膜转移患者脊髓病理改变, 从颈髓节段到骶髓节段的蛛网膜下腔均有肿瘤细胞弥漫播散, 肿瘤于软脊膜表面形成浸润灶, 甚至侵及脊髓实质, 病变多发生于白质的血管周围间隙, 肿瘤细胞亦可浸润于神经根, 导致相应的病理损坏。我们的研究中, 5例患者进行了腰椎穿刺, 细胞学发现瘤细胞, 提示广泛的蛛网膜下腔的肿瘤细胞播散, 是进一步软脊膜肿瘤细胞浸润转移的基础。

在肺癌中枢转移的诊治过程中, 对于临床症状的识别和分析至关重要。在肺癌临床诊断过程中, 颅高压表现(如恶心、呕吐、头痛等)以及颅神经受累表现(复视、听力下降等), 进而行头增强MRI及腰椎穿刺行脑脊液细胞学检测, 进而明确脑膜转移诊断。同样, 脊膜受累或脊神经受累的相关症状, 强烈提示软脊膜受累。在肺癌的硬膜下脊髓外转移的相关研究中, 马尾综合征为最常见的临床症状^[8, 9]^, 临床表现为腰骶部疼痛, 双侧或单侧坐骨神经痛, 鞍区感觉减退, 尿潴留、便秘, 下肢感觉减退或下肢运动功能减退等, 在本研究中, 马尾综合征亦为最为常见的临床症状, 8例患者均有不同程度的马尾综合征表现。1例患者有颈髓节段脊膜受累, 亦表现相应的典型的上肢疼痛及无力的表现。因此, 在肺癌患者中, 症状和查体对于临床决策的判断极为重要, 如常规病变不能解释的脊髓受累的表现, 应积极行进一步的脊髓增强MRI进一步检查。同样, 由于软脊膜转移是脑(脊)膜转移的一种临床表现, 部分患者合并脑膜转移的临床症状, 而其他的两类转移癌脊髓压迫综合征仅有脊髓压迫表现, 无脑膜转移相关表现。

增强MRI是转移性脊髓压迫综合征最敏感和最特异的影像学检测方法, 对于肺癌患者如出现脊髓及脊神经受累的症状体征, 需积极完善脊髓增强核磁检查。通过MRI, 可以整体观察脊柱、椎管、硬脊膜、蛛网膜下腔、脊髓和脊神经受累情况, 可以有效识别椎管内外结构病变, 鉴别病变位置及性质, 进一步鉴别硬膜外转移、髓内转移受累或是硬膜下脊髓外转移^[10, 11]^。对于椎管内硬膜下脊髓外转移的全脊髓增强MRI, 其影像学表现可表现I分为三种类型^[12-14]^:①硬膜下脊髓外结节样改变, T1WI等信号结节样软组织信号影, T2WI表现为蛛网膜下腔内大小不一的结节状充盈缺损, 增强扫描呈轻度强化; ②软脊膜增厚伴有线样增强, 极少数情况可见脊神经增强或增厚; ③混合型。对于合并有结节样改变的髓外病变, 易于识别和诊断。仅有软脊膜增厚伴有线样增强的病变, 如进行常规核磁, 难以识别, 而且即使行增强MRI, 如病变影像学改变程度轻, 需结合患者临床症状, 由放射科医师与临床医师共同讨论后方可诊断。本研究中3例患者仅表现为软脊膜的增厚及增强, 脑脊液中均找到瘤细胞, 经放射科医师与临床医师共同讨论后诊断脑(脊)膜转移。

治疗上, 不同于硬膜外压迫或髓内转移的手术或放疗为主的局部治疗方式, 软脊膜转移的肿瘤细胞, 在脊髓蛛网膜下腔形成多发结节转移灶或弥漫性软脊膜受累病变, 无法行手术治疗, 治疗困难, 需多学科综合治疗。由于发生脑(脊)膜转移后患者临床症状重, 无法耐受化疗, 因此, 系统治疗方面以靶向治疗为主。由于EGFR-TKIs及ALK-TKIs作为小分子药物, 可以一定程度透过血脑屏障, 尽管脑脊液浓度极低, 仍部分患者可能产生一定的效果, 多项研究报道EGFR-TKIs治疗*EGFR*突变的脑膜转移患者, 可延长生存期。有研究^[15, 16]^报道*EGFR*敏感突变的NSCLC软脊膜受累的脑膜转移癌患者, 吉非替尼治疗后硬膜下脊髓外多发结节明显缩小, 在我们的研究中, 1例*EGFR*突变患者软脊膜转移后应用EGFR-TKIs并加用局部马尾病变的放疗, 症状明显缓解, 其他3例EGFR-TKIs治疗过程中发生软脊膜转移或2例EGFR野生型患者, 应用EGFR-TKIs治疗效果欠佳。值得注意的是, 本研究中1例存在*ALK*重排的患者, 一代ALK-TKI克唑替尼治疗失败, 停用药物后, 发生软脊膜转移, 再次应用二代ALK-TKI色瑞替尼治疗, 症状及影像学均有缓解, 提示二代ALK-TKI可一定程度透过血脑屏障, 并可克服一代ALK-TKI耐药, 治疗脊膜转移可再次有效。鞘内注射化疗作为一种局部给药的治疗措施, 是一种传统的治疗脑(脊)膜转移的方法, 在Gwak等^[17]^研究中, 合并马尾综合征的13例患者, 经脑室内注射化疗后, 仅2例(15%)患者症状改善, 并有部分患者新出现马尾综合征, 其原因可能与鞘内注射药物对肺癌有效率差相关, 鞘内注射治疗的疗效尚需要进一步评估。放疗可以作为一种局部治疗的方式, 有明显局灶性病变的患者有改善症状的效果^[18]^, 在本研究中, 3例患者进行了局部病灶的放疗, 症状得到了不同程度的改善或症状稳定, 且生存期分别为5.8个月、7个月及14个月。对于本组研究的患者, 由于脊膜病变及脊神经病变受累相关症状, 该组患者生活质量差, 有效的治疗, 可能改善患者临床症状, 本组患者中位OS为5.8个月, 提示经过积极的综合治疗, 硬膜下脊髓外转移患者生存期有一定的改善。

综上, 硬膜下脊髓外转移是肺癌患者的灾难性转移事件, 马尾综合征是最常见的临床表现, 严重影响患者生活质量。全脊髓增强MRI是诊断的有效定性定位措施, 可表现为软脊膜弥漫线样增强和硬膜下脊髓外多发结节, 脑脊液细胞学检测找肿瘤细胞为阳性。靶向治疗和/或可能改善神经系统症状, 但预后仍很差。
